# Application of Neural Network Automatic Event Detection for Reservoir-Triggered Seismicity Monitoring Networks

**DOI:** 10.3390/s26030783

**Published:** 2026-01-23

**Authors:** Jan Wiszniowski, Grzegorz Lizurek, Anna Tymińska, Paulina Kucia, Beata Plesiewicz

**Affiliations:** Institute of Geophysics, Polish Academy of Sciences, 01-452 Warsaw, Poland; lizurek@igf.edu.pl (G.L.); atyminska@igf.edu.pl (A.T.); pkucia@igf.edu.pl (P.K.); bples@igf.edu.pl (B.P.)

**Keywords:** reservoir-triggered seismicity, seismic event detection, seismic network, time-series analysis, phase association, deep neural network

## Abstract

**Highlights:**

**What are the main findings?**

- The study found that manual and various automated seismic detection methods yield disjointed sets of events.
- Thresholds for detecting weak events recorded by a small number of stations result in a significant number of false detections or incorrect picks; however, detection sensitivity was found to be stable as the RTS evolves.

**What are the implication of the main findings?**

- The key implication of these findings is that no single seismic signal detection method is adequate.
- Therefore, we propose combining various detection methods to enhance the overall effectiveness and accuracy of seismic event analysis related to reservoir-triggered seismicity.

**Abstract:**

This study examines reservoir-triggered seismicity (RTS) in Poland and Vietnam. The current state of individual RTS seismic networks necessitates detecting earthquakes from only a few stations. The number of P waves is often inadequate for phase association and event location, which underscores the importance of identifying S waves. Given that individual RTS cases may consist of only hundreds of events, it is crucial for algorithms to be trained on small datasets or to detect effectively using external, global training data. To evaluate this, we compared the efficiency of a deep learning global detection model, transfer learning to the RTS database, a specialized neural network designed for RTS, and manual detection of seismic signals. Transfer learning efficiency was database dependent. Additional interpretation and parametrization of detection results are assumed. Therefore, the emphasis is on phase detection, rather than phase picking accuracy, and detection sensitivity is more important than its specificity. Phase association plays a vital role in detecting seismic signals, facilitating the elimination of most false picks. As a result, the comparisons of detections were based on parameters related to the location of seismic events. The findings indicate that neither the automatic signal detection methods nor the manual methods alone are sufficient. However, their combination significantly enhances detectability. The final catalogs cover up to 30% more events compared to the previous manual. It fulfills the main aim of applying a neural network detector, which is to increase the number of seismic events in the catalog. It may also be further utilized in the research of the triggering process, such as identifying fluid paths and determining fault geometry.

## 1. Introduction

Water impoundment in artificial reservoirs plays a crucial role in providing low-emission and stable energy production, and the global demand for hydropower continues to grow. Among other countries, Vietnam is significantly expanding its hydropower plants. However, this practice is associated with reservoir-triggered seismicity (RTS), which has become a significant area of research. Artificial reservoirs often trigger anthropogenic seismicity due to changes in pressure and stress on the Earth’s crust [1]. Carder [2] was the first to point out the connection between earthquakes and Lake Mead’s artificial water reservoir in the United States. Since then, RTS cases have continued to increase [1]. These events can range from minor to significant earthquakes, and seismic responses can change over time. The largest reservoir impoundment earthquakes are damaging, like the magnitude 6.3 earthquake at Koyna Dam, India, in December 1967, which caused substantial damage [3]. Such strong events are especially damaging in areas of low natural seismicity due to the lack of detailed seismic research and earthquake-resistant infrastructure, including dams. The case of Koyna Dam is now well documented and has been studied extensively using a dense seismic network. However, many reservoirs worldwide produce felt seismic events that do not generate enough public interest to warrant in-depth studies with extensive seismic monitoring. As the RTS potential for damaging events differs widely depending on the tectonic regime as well as geological and hydrological conditions, each case should be examined carefully to understand the complexities of RTS mechanisms, both generally and locally [4,5,6,7]. For a better understanding of the mechanisms driving seismicity, high-quality and large databases are required. Networks with fewer stations typically monitor these reservoirs. Yet, such data are the only available option to investigate the influence of the reservoir on seismicity changes and risk mitigation in these cases [3,8]. The number of stations is often inadequate for phase association algorithms. Therefore, for automatic event detection, the accurate single-station phase detection is crucial.

Reservoir impoundment is well known for its capability of triggering damaging earthquakes [1], but every new reservoir needs to be considered separately in view of seismic hazard and its mitigation. Seismic monitoring provides data from the investigated area, rather than regional data typically available from national seismic networks. The latter may be too sparse and/or have insufficient spatial resolution, as well as a completeness magnitude threshold that can be too high for anthropogenic hazard assessment. The Department of Seismology at the Institute of Geophysics, Polish Academy of Sciences (IG PAS), collaborates with the Institute of Geophysics, Vietnam Academy of Science and Technology (IGP VAST) to monitor and study RTS in various locations, including Czorsztyn in Poland [8,9], Song Tranh 2 [10,11], and Lai Chau in Vietnam [12]. These are examples of RTS with sufficient diversity of both seismicity and monitoring conditions for detection studies. Vietnam is particularly prone to RTS due to significant investments in hydropower plants, especially in northern Vietnam, where moderate regional seismicity is continuously observed, and several dams were built in this area. The strongest recorded RTS earthquake, a magnitude 4.9, appeared in Hoa Binh Province, Northern Vietnam [13]. Unfortunately, this area was not monitored by an appropriate seismic network.

Traditional methods of detecting seismic events and picking phases rely heavily on manual analysis, which limits their efficiency and accuracy when processing large datasets. As a result, the manual interpretation of all RTS signals frequently exceeds the capabilities of the research institutes involved. Seismicity characteristics can vary significantly among different RTS cases. In certain situations, detecting minor events is crucial for gathering sufficient research data. In contrast, natural seismicity complicates the analysis in others, necessitating a clear distinction between natural and reservoir-induced events. This complexity underscores the need for advanced monitoring and detection methods to enhance the understanding of RTS. As the complexity of RTS leads to a wide variety of events recorded under specific conditions, we initially assume that automatic detection alone is insufficient for both determining whether an event occurred and parameterizing it. Therefore, each automatically detected event requires manual verification and parameterization (e.g., location and magnitude calculation), and the accuracy of automatic parametrization is neglected in this study. The development of automatic signal detection began in the late 1970s, with the advent of digital recording. Withers et al. [14] categorized the initial detection methods into four types: time domain, frequency domain, particle motion processing, and pattern matching. Time domain methods rely on envelope analysis and comparing short-term and long-term STA/LTA ratios [15,16], and they are in use today [17]. However, these methods and earlier machine learning techniques [18,19,20] are sensitive to background noise. In the first decade of this century, three synchronous trends emerged, transforming the approach to machine learning: the availability of Big Data, the reduction in the cost of parallel computing, and the introduction of new deep learning algorithms. Without sufficient data, the training can lead to overfitting, resulting in a loss of generalization. The sets used for training seismic detection consist of millions of waveforms [21,22,23,24,25]. It has been observed that DNN detectors frequently exhibit a decline in performance in various seismic monitoring scenarios [26,27,28]. The available RTS training sets are significantly smaller. Transfer learning is an attempt to solve this problem [28,29]. However, the generalization of transfer learning to anthropogenic seismicity, which varies over time, requires further study.

This research aims to investigate the effectiveness of DNNs, including transfer learning, in detecting seismic RTS signals. Specifically, the study seeks to determine whether it is feasible to automate seismic signal processing using DNNs and whether this approach can enhance the investigation of the causes of RTS by increasing the number of analyzed tremors. In the presented work, the non-stationary nature of RTS is investigated to determine how the selected training database affects detection in other periods. By leveraging the capabilities of DNNs, the research aims to improve detection accuracy and efficiency, ultimately providing deeper insights into seismic events and their underlying mechanisms. Therefore, the goal of this study is not to fully automate seismic data processing. Additional interpretation of detection results is assumed, so the emphasis is on the phase detection, not exact picking, and the detection sensitivity (recall) is more important than its precision.

## 2. Materials and Methods

### 2.1. Investigated Seismic Networks

The density of the RTS monitoring seismic network is crucial for the automatic detection of seismic signals. There are many areas of RTS around the world, but seismic networks in these areas are usually temporary or have a sparse number of stations, mainly before the triggering occurs. We decided to use datasets acquired with reasonably dense seismic networks that have been operational for longer than several months and located in different tectonic areas to test the proposed methods. The seismicity in the studied area is the second criterion, alongside the configuration of the monitoring network, which defines the requirements and possibilities for automatic detection. Seismic conditions and tectonic settings in the studied regions vary significantly. The STr2 region had variable station setups. A long-term monitoring campaign was conducted in this area, situated in a stable tectonic setting where triggered seismicity prevails over natural seismicity [11]. On the other hand, the Lai Chau area is dominated by moderate magnitudes and regional tectonic events with a small fraction of triggered seismicity [12]. The third site is considered the main tectonic boundary area between two major Alpine orogeny units—the Inner and Outer Carpathians, with sparse seismic activity despite the active tectonics described [8]. Such a site’s choice allows us to test the proposed methods in different environments, in terms of station setup, seismic activity rate, and their magnitude range.

#### 2.1.1. Song Tranh 2

**Figure 1:**
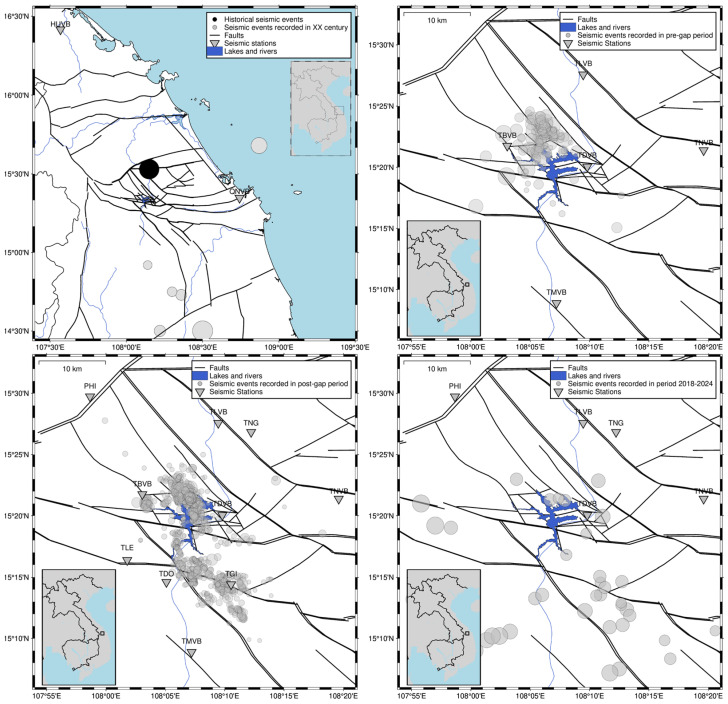
Song Tranh network changes in time: stations’ locations before the impoundment of the lake (**top left panel**), pre-gap period 2012–2013 (**top right panel**), post-gap period 2013–2017 (**bottom left panel**), and actual network after 2022 (**bottom right panel**). Stations are denoted as triangles with station code above, and black solid lines denote main tectonic discontinuities in the area.

The monitoring campaign consisted of four stages with varying numbers of stations, including 10 local stations deployed in the vicinity of Song Tranh 2 (STr2) (Figure 1). Four stages correspond to the reservoir filling periods and seismic activity in the area: the first, a period of initial impoundment, hereinafter referred to as the pre-gap period (from 5 January 2011 to 10 June 2012), then the second, a gap period (from 10 June 2012 to 31 August 2013), during which reservoir impoundment stopped and water was drained to minimum exploitation level, the third, the post-gap period (from 31 August 2013 to 19 June 2017), and the fourth, the seismic quiescence period, which covers the time after 2019, when rearrangement of stations due to the substantial decrease in seismicity took place, which resulted in decreasing of station number to seven and further to five. The spatial distribution of events and stations in every phase is shown in Figure 1. Before and just after the beginning of filling the STr2 reservoir (pre-gap period), the area was monitored by two seismic stations in Binh Dinh and Hue, located away from the reservoir (situated outside the Song Tranh 2 lake area—Figure 1). It makes these recordings unusable for automatic processing. The more precise monitoring of triggered seismicity in the STr2 reservoir was installed from October to November 2012, when IGP VAST deployed a five-station network in the STr2 reservoir and its vicinity. They were equipped with Guralp 30 s seismometers connected to the SAMTAC loggers and one Trillium-40 seismometer with the Q330 logger. The third stage of the monitoring started in August 2013, when 10 seismic stations were installed. This seismic network was called VERIS (Vietnam Reservoir Induced Seismicity). Stations provided by IG PAS were equipped with short-period seismometers (Lennartz LE-3DLite, 1 s), while those provided by IGP VAST were equipped with long-period seismometers (Guralp CMG-6TD, 30 s). Signals from the Lennartz seismometers were recorded at a sampling rate of 100 samples per second and with a dynamic range of 132 dB. The Guralp seismometer features an onboard digitizer with a dynamic range of 130 dB and a sampling rate of 100 samples per second. Both systems are suitable for measuring local and regional seismicity, as the main frequency content of seismic waves falls within the range of a few Hz. It should be noted that even during the post-gap period, not all stations operate continuously, which makes automatic detection more challenging in cases of weak events due to variable station numbers and setups. Therefore, the analysis and detection of seismic signals were performed manually.

Central Vietnam, where Str2 is located, is characterized by very low natural seismic activity. Only 13 events in this area have been reported historically, and only one earthquake (shown in Figure 1), with a magnitude of 4.7 in 1715, determined based on seismic intensity, was located near the reservoir [30]. Despite this, we recorded the strongest RTS of all the tested areas (Table 1). The seismicity is grouped into two main clusters, located in the north and south of the reservoir, as well as a few smaller clusters. The clusters differ in magnitude distribution, focal mechanisms, and temporal variation in seismicity [11]. The seismic activity in the south cluster appeared just after impoundment, whereas the seismicity in the north cluster was delayed, which may indicate a combination of short- and long-delay response type of seismicity [3,11].

#### 2.1.2. Lai Chau

**Figure 2:**
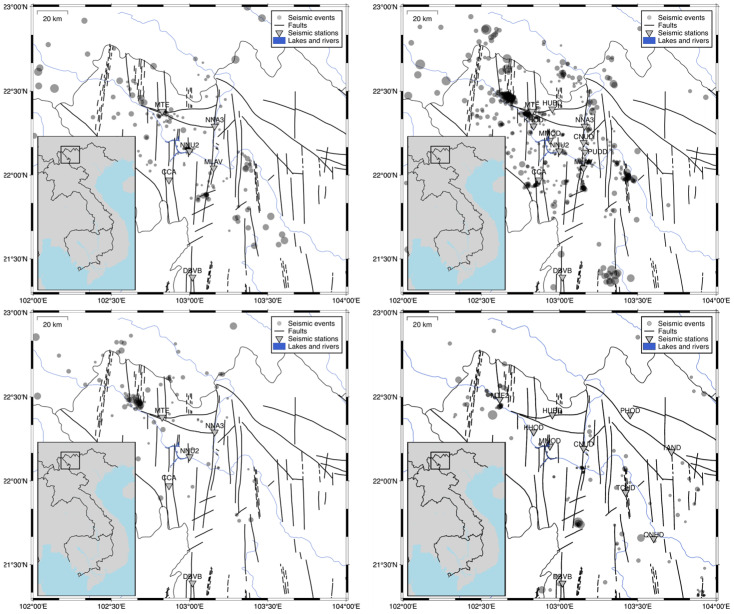
Lai Chau network changes in time with seismic activity above M1: 2015 before the impoundment of the lake seismic activity in period 2014–2016 (**top left panel**), full deployment with seismic activity in period 2017–2020, (**top right panel**), reduced network after 2022 with seismic activity in period 2021–2022 (**bottom left panel**), upgraded actual network after 2023 with seismic activity in period 2023–2024 (**bottom right panel**). Stations are denoted as triangles with station code above, and black solid lines denote main tectonic discontinuities in the area.

We planned to monitor the area around the Lai Chau dam using a local network of at least ten seismic stations. The network was developed gradually. Initially, the Vietnamese National Stations Network monitored the seismicity of the Lai Chau region. There were seven stations in North Vietnam, with Điện Biên (DBVB) station 80 km from the Lai Chau region. The Sa Pa (SPV) station, which has been operating since 1957, was located 90 km from the area, too far to be included in the network. Only the station in the Mường Lay (MLAV) was located in the Lai Chau region, 16 km from the dam (Figure 2). The IGP VAST set up several local stations dedicated to monitoring the area of the Lai Chau reservoir. Three stations in Nậm Nhùn (NNU), Chà Cang (CCA), and Mường Tè (MTE) were installed in September 2014, and one station, Nậm Na 3 (NNU3), was installed at the beginning of the reservoir impoundment in June 2015. The following stations in Chăn Nưa (CNUD), Pú Đao (PUDD), Kan Hồ (KHOD), and Hua Bum (HUBD) were launched by the IG PAS in July 2016, after the reservoir had been filled. The last station in Mường Mô (MMOD) was activated in June 2017. In 2022, the network was reduced to five stations, including the DBVB broadband station, due to low activity in the area and cost optimization. In late 2023, the network was upgraded to monitor a broader area along the Da River. Figure 2 presents the locations and parameters of the stations. Very broadband, broadband, and short-period sensors were used. All stations have a sampling rate of 100 samples per second. The positions of stations are determined by the location of previous seismic events and by a desire to provide good horizontal coverage for events around the dam. It should help estimate the moment tensor of reservoir-triggered events more accurately. However, the station’s location was strongly limited by logistical possibilities: poor urbanization and a small number of roads in this area.

Additionally, stations had to be located in residential areas for security reasons. Therefore, the western part of the reservoir is poorly covered with stations (Figure 2). The detailed location of the stations was preceded by ground-penetrating radar measurements to identify the foundations of the stations on the bedrock. It allowed for the reduction in both natural and human noise and improved the detection of seismic events. It should be noted that not all stations were operational at all times. Despite this, the signal analysis was supported by SLRNN detection, which was specifically developed to work with a poor seismic network.

The Lai Chau dam and reservoir are situated in an area with fault zones associated with natural seismic activity [12]. This region experiences moderate seismic activity, with the strongest earthquakes reaching magnitudes of up to 5.6. The monitoring network was established to study changes in natural seismicity, cataloging events that occurred before the reservoir impoundment, as well as those triggered by water impoundment. Various seismic events are observed in the Lai Chau region, which influence the shape of the recorded signals.

#### 2.1.3. Czorsztyn

**Figure 3:**
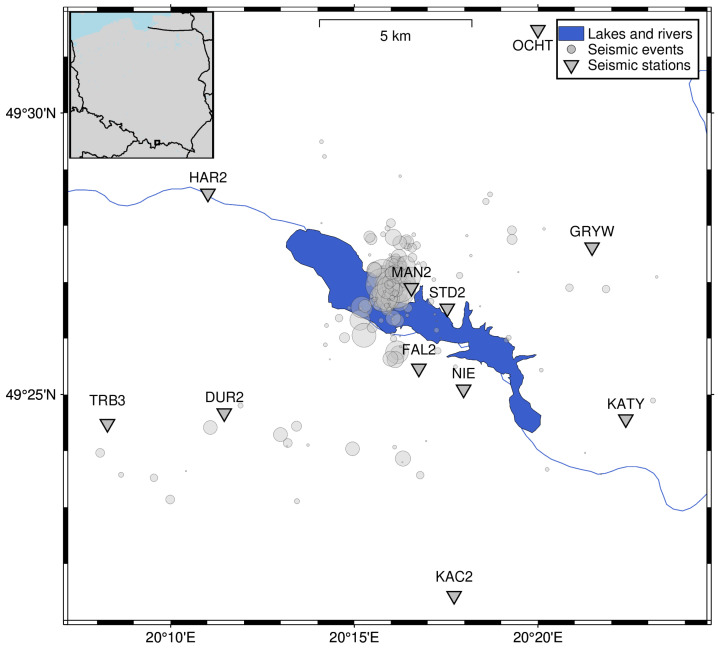
SENTINELS network and NIE broadband station locations around the Czorsztyn Lake. Stations are denoted as triangles with station code.

Seismicity measurements due to increased seismicity rate near Czorsztyn Lake (since 2011) started in August 2013. IG PAS, in cooperation with Niedzica Hydro Power Plants Company (ZEW Niedzica S.A., Nowy Targ, Poland), launched a local seismic network, SENTINELS (**SE**ismic **N**etwork for **T**r**I**ggered and **N**atural **E**arthquake **L**ocation and **S**ource determination). The first network configuration contained seven short-period Lennartz 3D Lite seismometers with NDL recorders (Figure 3). The network was upgraded at the turn of 2013 during the IS-EPOS project (IS-EPOS: Digital Research Space of Induced Seismicity for EPOS Purposes). Lennartz equipment was replaced by GeoSig short-period (5 s) VE-53 seismometers with GMSplus3 recorders. Due to the noise, one station was relocated, and three new stations were founded. Since then, the network has consisted of 10 short-period stations, located around Czorsztyn Lake in facilities of ZEW Niedzica S.A., the Krościenko administration of forestland, the Nowy Targ municipality, and private land. The short-period stations were supported by the broadband station NIE from the PLSN (Polish Seismological Network), equipped with an STS-2 very broadband seismometer. All stations have a sampling rate of 100 samples per second. SENTINELS seismic network has recorded almost 300 seismic events from August 2013 to January 2022 [8,9]. Figure 3 shows the locations of stations from the end of 2013 to 2025. The NIE station and 10 short-period stations proved to be efficient for observing triggered seismicity around Czorsztyn Lake [8] and as an addition to regional tectonic activity observations [31,32]. It is worth mentioning that this network operated consistently throughout the entire period, with minimal gaps and interruptions in station operation. Conversely, the installation of most stations within the dam and hydropower plant infrastructure leads to higher background noise measurements compared to other seismic networks. The analysis of seismic signals and the detection were performed manually, which is now replaced by automatic earthquake detection and processing.

The reservoir in Czorsztyn is situated on the Pieniny Klippen Belt, which is characterized by weak and sparse seismicity (Table 1), although a historical earthquake with a magnitude of 5.5 was recorded. In the vicinity of the reservoir, seismicity in Slovakia and seismicity induced by coal mining in Upper Silesia, Poland, influence the automatic detection. Many years after the impoundment in 1997, no seismic activity was noticed, and only the station in Niedzica monitored the reservoir. Then, we observed the swarm seismicity, first in November and December 2011, before the local network was put into operation. The distant network monitoring the Podhale seismic active area detected almost one hundred earthquakes with magnitudes ranging from 0.5 to 2.2 [33]. The seismic network SENTINELS monitored the following seismic activity.

### 2.2. Automatic Detection of Reservoir-Triggered Seismic Signals Methods

Automatic seismic signal detection encompasses automatic seismic wave detection or phase picking methods, which are treated here as detection, as the picking accuracy plays a lesser role in this context. All detection algorithms detect a large number of false waves, so the second necessary step is phase association to eliminate false detections. In real-world RTS monitoring conditions, this is difficult due to the insufficient number of intermittently operating seismic stations.

The seismic stream preprocessing, including filtering and normalization, is integrated into the detection algorithm. However, we do not prefer the signal-to-noise filtering, which removes seismic signal differences from disturbances and makes noises similar to seismic waves [34].

#### 2.2.1. Single-Layer Recurrent Neural Network

A single-layer recurrent neural network (SLRNN) was initially developed to detect earthquake swarms in West Bohemia [35]. It was modified by incorporating new inputs and multi-stage training integrated with interpretation and applied to detecting natural and reservoir-triggered seismic events in the region of the Lai Chau dam and reservoir [36]. It is a purely recurrent neural network where the detection input is the current seismic signal. Instead of a sliding time window, the temporal relationships of signals are stored in recurrent neurons. The initial real-time recurrent neural network, consisting of a single layer of recurrent neurons, was developed to detect seismic signals of diametrically different lengths, from teleseismic signals to local ones [37]. However, it had a drawback: it tended to forget information about previously detected seismic waves—especially distant and teleseismic signals—before the next ones arrived [34]. This issue was handled by Doubravová et al. [35] through the use of a modified Nonlinear Autoregressive Neural Network [38].

**Figure 4:**
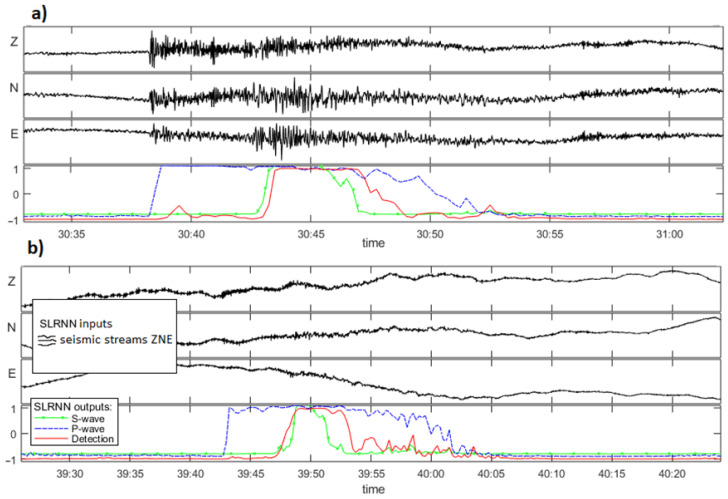
Results of signal detections by the SLRNN [36], (**a**) an average event and (**b**) a weak event at noise level.

The advantage of this network was its independence from the duration of the seismic signal. Training such a neural network for extended periods proved challenging, although it possessed a notable degree of freedom that facilitated relative learning with small datasets. This solution also demonstrated satisfactory generalization capabilities [36]. The SLRNN detects seismic waves and events at individual stations. However, due to its design, it cannot automatically pick seismic phases. The P and S wave detection is intentionally delayed so that the neural network collects information about the signal after the wave input (Figure 4). The output of the P wave is trained to remain high until the onset of the S wave, facilitating the multi-phase identification of the seismic event. The delay in wave detection depends on the signal. The SLRNN supports manual analysis of seismicity by detecting potential seismic signals.

#### 2.2.2. Deep Neural Networks

The detection of seismic phases was performed using the SeisBench program developed by Woollam et al. [39]. This open-source package is designed to help users apply machine learning techniques to seismic data. The software provides access to recently published comparative datasets for machine learning in seismology, which can be easily downloaded and utilized. SeisBench further enhances this concept by offering a unified access point to machine learning models, including state-of-the-art models and their corresponding weights for seismic tasks. We tested several models, including:

- Basic Phase Auto-picker (BasicPhaseAE) by Woollam et al. [40], based on a U-Net type convolutional neural network (CNN).
- EarthQuake Transformer (EQTransformer) [41], which combines CNN and long short-term memory recurrent neural networks.
- Generalized Seismic Phase Detector (GPD) [42], based on a ConvNet type CNN,
- PhaseNet [43], based on the U-Net and computer vision.

For detection training, we selected datasets with a significant number of local events having magnitudes less than one, as the current completeness magnitudes of RTS catalogs are below 1. We also recorded events with magnitudes less than 0. Additionally, the datasets should exclude distant events, as some RTS areas are located in regions of moderate seismicity, and we aim to avoid detecting natural earthquakes. The models were pre-trained on three seismic datasets (Table 2):

- LENDB: This benchmark dataset, created by Magrini et al. [44], includes labeled seismograms from local earthquakes and noise recorded by numerous seismic receivers worldwide. It has a large number of elements, but the limited number of S picks restricts phase association and location accuracy due to the small number of stations.
- SCEDC: The Southern Californian Earthquake Data Centre dataset contains the most events and P and S picks [45]. However, it lacks examples of noise, which may affect precision.
- STEAD: This Stanford earthquake dataset contains many events, P picks, S picks, and noise [46].

The PhaseNet model, pretrained on the SCEDC and STEAD datasets, was used for transfer learning by the SeisBench program.

#### 2.2.3. Phase Association

In some cases, certain combinations of deep learning models and datasets generate an excessively high number of detected picks, necessitating their reduction. Two primary methods are used to manage seismic signal and event detection: setting a minimum peak value of the detection characteristic function, often referred to as pick probability, and specifying the number of seismic phases that must be present during event association. During the selection of minimum peak values and the number of seismic phases aimed to maximize sensitivity, which was evaluated by comparing the number of events detected with phase association to those identified manually. We empirically tested peak value ranges from 0.1 to 0.9, setting the required numbers for both P and S phases from 2 to the number of stations. While lower values increase sensitivity, specificity values tend to be unrealistically high. Conversely, higher peak values and larger required numbers suggest an unacceptable low sensitivity compared to manual detection.

Additionally, some required pick thresholds cause detection gaps when the number of operational stations decreases. The lack or misassociation of S-wave detection completely prevents phase associations in such cases. Based on the test results, the chosen values do not achieve 100% sensitivity but enable human review of all automatic detections more effectively than continuous recording analysis. These values were different for each RTS case and were selected individually. Two seismic association methods for phases were tested: PhaseLink [47] and PyOcto [48]. The velocity model inaccuracies were considered, and the phase association tolerance was determined based on the pick residuals of the previous event locations.

### 2.3. Detection Efficiency Assessment Methodology

Detection efficiency is assessed by comparing automatically detected events with a reference catalog of previously processed events, mainly derived from manual signal interpretation of seismic networks. The catalog created through automatic phase picking, event association, and localization is compared with the reference catalog in QuakeML format. This comparison yields three outcomes: catalogs of automatically detected events, undetected earthquakes from the reference catalog, and additional detections not found in the reference catalog, which may include both false detections and real earthquakes. The extra detections in several selected periods and detection settings are manually evaluated using the SWIP5 program to classify the types of detections and assess their proportions, thereby evaluating the detection algorithm. Finally, extra earthquakes are analyzed and added to the catalog if possible.

**Figure 5:**
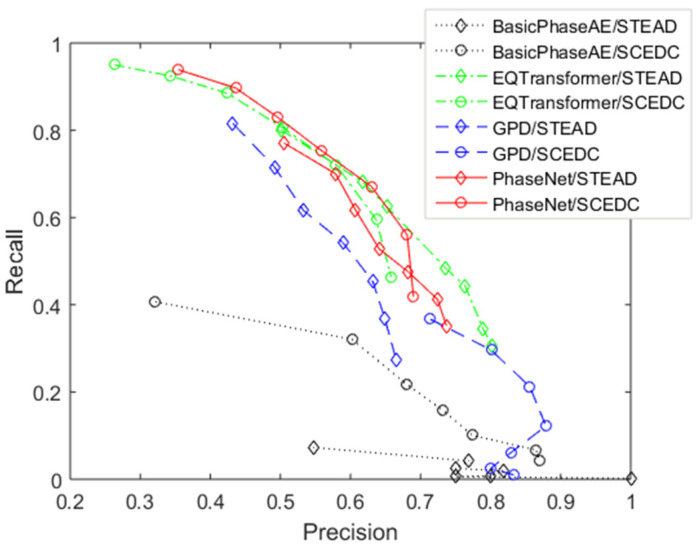
Recall-precision diagram of various deep learning seismic detection models in VERIS network in STr2 detections in 2013 and PhaseLink association.

**Figure 6:**
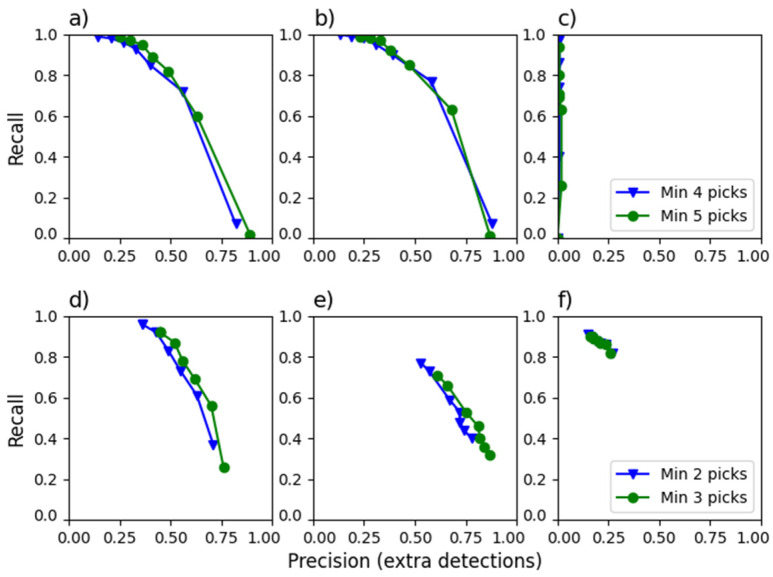
Recall-precision diagrams for various pick detection values (0.3–0.9) and two minima of required phases for association by PyOcto: (**a**) STr2 2013, EQTransformer model, SCEDC dataset; (**b**) STr2 2014, EQTransformer model, SCEDC dataset; (**c**) STr2 2018, EQTransformer model, SCEDC dataset; (**d**) STr2 2013, PhaseNet model, SCEDC dataset; (**e**) STr2 2013, PhaseNet model, STEAD dataset; (**f**) STr2 2013, SLRNN. In (**a**–**e**), four (triangles) and five (circles) phases are required. In (**f**), two (triangles) and three (circles) phases are required, because SLRNN was developed for a small number of stations.

**Figure 7:**
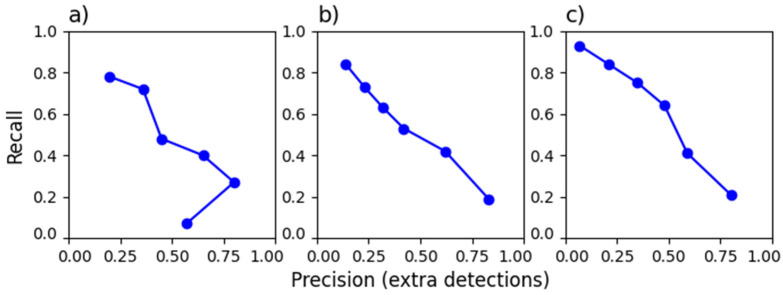
Recall-precision diagrams for various peak values of the EQTransformer model with SCEDC dataset detection characteristic function and minimum picks of PyOcto phases association (on the left), and the dependence of recall and F1 score on peak value (on the right), for various RTS monitoring years at Lai Chau under changing seismic network conditions: (**a**) 2014; (**b**) 2016; (**c**) 2018.

To assess detection efficiency, we apply commonly used [26,41,43] key metrics such as true positive (TP), false positive (FP), false negative (FN), and true negative (TN), along with derived evaluation parameters like precision (P = TP/(TP + FP)), recall (R = TP/(TP + FN)), and F1 score (F1 = 2PR/(P + R)). Recall (also called sensitivity), precision, and F1 score depend on the minimum value of picks and the minimum number of phases in association. Figure 5, Figure 6 and Figure 7 present these dependencies. It would be ideal to achieve high recall and precision values, close to 1. Unfortunately, they behave oppositely. For this reason, the F1 score is used as a balance between them. On the other hand, recall is the most crucial metric when the goal is to avoid missing events, as in the RTS cases studied.

We lack complete information about all events, as reference catalogs and manual signal analysis do not include all expected seismic events. Many events were not recorded in the catalog due to insufficient picks. As a result, the TP identified through comparison with the reference catalog is likely lower than the actual TP. Conversely, what are classified as false detections, or extra detections, may include real seismic events that are not present in the reference catalog. Therefore, the false FP obtained from this comparison will also be underestimated. Consequently, we should assume that the actual recall and precision are higher than those derived from the reference catalog, and we should take this into account when evaluating the results.

## 3. Results

### 3.1. Preliminary Selection of Seismic Signal Detection Algorithms

For further study, we selected the most promising deep learning model, the dataset for pre-training, and the phase association algorithm. For comparison, we focused on the initial four-month registration period of RTS on STr2 in 2013, when the network was nearly fully operational. We chose STr2 due to its high seismic intensity. A detailed manual analysis identified 447 events with magnitudes ranging from 0 to 3.4. However, the data collected in the studied RTS areas (Table 1) was insufficient for deep learning applications. As a result, we had to utilize publicly available benchmark datasets (Table 2).

Phases were subsequently associated using the PhaseLink program. We tested minimum phase requirements ranging from 4 to 14, as fewer stations often record minor earthquakes. Models trained on the LENDB dataset were found to be ineffective, primarily due to the absence of S picks in the training set, which limited their performance in smaller networks. Figure 5 presents the recall-precision diagram of detections based on the minimum required number of seismic phases. The relative detection precision of models trained on the STEAD dataset surpassed that of models trained on the SCEDC dataset, likely due to the lower noise levels in the SCEDC data. A comparative analysis for STr2 reveals that the GPD and BasicPhaseAE models performed less effectively, while EQTransformer and PhaseNet exhibited comparable results. One model trained on a single dataset was employed for further studies on specific seismic networks, including PhaseNet, trained by STEAD, and EQTransformer, trained by SCEDC.

### 3.2. Detection Based on the SENTINELS Network in Czorsztyn

For the SENTINELS network, PhaseNet signal detection trained on the STEAD dataset was utilized, and its performance was evaluated based on specific criteria:

- Phases detected with a minimum peak value of 0.367 were considered valid.
- Phase association was conducted using the PyOcto algorithm.
- The association required a minimum of 5 picks, including at least 2 P and 2 S picks.

The evaluation against manual detection revealed that the algorithm failed to detect 29 events with magnitudes between −0.7 and −0.6, resulting in a recall of R = 0.90. Analysis of the undetected events indicated that the NIE broadband station, part of another network (PLSN) [49], played a crucial role in detecting seismic waves, even at low amplitudes. This station was not included in the detection process, which likely affected the identification of low-magnitude earthquakes. The stations’ channels within the SENTINELS network were quite noisy, making it challenging to observe seismic signals without proper filtering. Some picks may have been detected manually in a distinctive manner, which could influence the identification process. Between 2013 and 2024, 590 automatically detected events around the Czorsztyn Lake, which were not included in the catalog, were analyzed and categorized into four groups:

1. Seismic Events: This group, comprising 41% of the extra detections, included phenomena classified as seismic events that were automatically picked near the lake. However, the locations had considerable errors, and manual verification showed that S-wave picks were often identified too early. The mentioned events were initially mislocated; however, manual correction enabled the determination of an accurate location. Some of these earthquakes were also recorded in other databases for the Upper Silesia, Podhale, and Slovakia seismic regions.
2. False Detections: This category accounted for 30% of the extra detections and included disturbances that did not exhibit characteristics of seismic signals.
3. Difficult-to-Classify Signals: Up to 14% of detections consisted of signals observed at one or two stations, rendering verification impossible due to the lack of broader recordings.
4. Distant Seismic Events: The last category comprised seismic events located approximately 20–30 km from the lake, accounting for 15% of all analyzed detections.

Overall, most detected tremors in the area were weak, small-amplitude events visible at only a few stations, making them difficult to notice during manual analysis. Comparing the years studied, such as 2020 to 2022, indicated a gradual decrease in activity, which may have affected the alertness of analysts who did not expect sudden increases in seismic activity. Additional detections often occurred during high-activity periods or after prolonged periods of inactivity, which can lead to the missed detection of minor shocks. To confirm these detections, further analysis, including correcting picks, adding more stations, and filtering data, would be necessary for clearer records.

### 3.3. Detection Based on the VERIS Network in STr2

In STr2, seismic signals were detected using the SLRNN trained on Lau Chau signals, the EQTransformer trained on the SCEDC dataset, and PhaseNet trained on the SCEDC and STEAD datasets. To reduce false seismic phase detections and accurately locate earthquakes, the PyOcto tool was employed. Manual detections were the reference for evaluation. Figure 6 presents the precision-recall (P-R) diagrams of the automatic detection results, using a pick probability threshold ranging from 0.3 to 0.9 and minimum phase limits for association of 4 and 5. The studies cover 2013 (Figure 6a), 2014 (Figure 6b), when most network stations were operational, and 2018 (Figure 6c), when the seismic network was functioning intermittently. The STr2 seismic network was installed in August 2013, leading to a four-month registration period. The records in 2013 were thoroughly examined, as all stations were operating at that time. Therefore, EQTransformer (Figure 6a), PhaseNest with SCEDC (Figure 6d), and STEAD (Figure 6e), as well as SLNNN (Figure 6d), were tested in this period. Two sets of extra detections by EQTransformer were selected for detailed analysis:

- A limit of 0.6 of the peak value of the detection characteristic function (short: peak value) with l an association with limit of 5.
- A peak value limit of 0.5 with an association limit of 7.

In the first case, 613 extra events were detected, of which 579 were identified as STr2 earthquakes. However, most events were correctly detected on only one or two stations, while false phases were identified on others. Although these events were manually recognized, two stations are insufficient for accurate localization and analysis, with fewer than 30% of events having sufficient signals for proper elaboration. In the second case, 298 earthquakes were found among 300 extra detections, with over 80% occurring in August and September. The number of events missing in the reference catalog may be attributed to the initial monitoring period. An optimal peak value threshold of 0.3 appears sensible, as the goal is to maximize the detection of weak events, even if this requires additional effort to eliminate false detections. Lower thresholds lead to an overwhelming number of false detections, necessitating manual analysis of continuous signals, while higher threshold values risk omitting significant phenomena. Notably, at a threshold of 0.9, even the strongest event, M = 3.6, in STr2 was not detected, and lower thresholds failed to identify events with magnitudes of 2 and above.

The application of PhaseNet yields comparable results (Figure 6d,e). It generates higher peak values, and the precision is better, especially in the case of the STEAD dataset. Therefore, for further analysis, we chose EQTransformer.

In the case of the SLRNN (Figure 6f), the limit of the pick threshold does not play as significant a role as it does in automatic picking, because of the training method of the SLRNN, where the shape of the output signals is more important than the specific values. As a result, many detections reach a value of 1 (e.g., Figure 4). In a limited number of cases, the corresponding seismic waves may not achieve this level. Still, the threshold’s influence on detectability is not as pronounced as for deep learning automatic pickers. Conversely, lowering the threshold significantly increases the number of false detections. With a threshold of 0.9, the SLRNN generated 1078 extra detections. Among these, 34% were false detections, 31% were seismic events visible at one or two stations, which hindered their localization, 28% were the new events that could be accurately located and processed, and 7% were regional and teleseismic events. False negative detections primarily involved the weakest signals, with magnitudes ranging from 0.1 to 1.1.

Because the seismicity in STr2 is higher than in other reservoirs, we applied transfer learning to the STr2 case to test whether it can improve detection. The primary question was whether the modified detection model would function correctly for future events, because seismicity in STr2 is not stationary. The seismic activity appeared in other clusters with other source mechanisms. To answer this question, we retrained the PhaseNet models, which were pretrained on SCEDC and STEAD, on seismic events manually detected in 2013. We then applied the transferred model to continuous recordings from 2013, 2014, when most stations were operational, and 2018, when the number of working stations was limited. Tests on data not used for transfer learning demonstrated that new models exhibit good generalization in the case of non-stationary RTS (Table 3). Transfer learning from the global STEAD model improved the recall, while transfer learning from the global SCEDC model did not. The reduction in precision caused by transfer learning does not automatically lead to more false detections, as many seismic events were not manually reviewed.

### 3.4. Detection in the Lai Chau Area

Since 2015, the SLRNN has been used to detect seismic signals in Lai Chau, followed by manual correction and interpretation. Signals from 2014 and 2015 were utilized as a training dataset for the SLRNN. Consequently, the catalog used to evaluate EQTransformer detection included only signals identified by the SLRNN. We analyzed three years of seismic signals to assess the effectiveness of EQTransformer detection (Figure 7).

The first period spans 2014 (Figure 7a), before the reservoir impoundment, when the network consisted of up to 5 stations and recorded natural background seismicity. The reference catalog contains manually detected events. The second period spans 2016 (Figure 7b), following the impoundment, during which SLRNN supported event detection. The third period spans 2018 (Figure 7c), during which the network expanded to 10 stations, capturing both natural and triggered seismicity. Most detections were local events, with very few false detections (Table 4). Some detected events, such as regional earthquakes or signals that cannot be definitively classified as earthquakes, are categorized as others. Many of the extra detections are weak earthquakes with magnitudes below 1. These events were only registered at nearby stations, and their signals were short and challenging to distinguish from noise. As a result, it is often impossible to parametrize such events. About half of the local events from 2018 were recorded at least on four stations, making them parametrizable. In contrast, in 2014, such events accounted for only 10–30% of local detections, depending on the thresholds used. In most cases of additional detections, only one or two stations recorded both P and S waves. For detections requiring at least 3 phases, most events were identified without any stations having both P and S waves picked, indicating that detection relied primarily on a single phase from a particular station. Among many events that, based on the signal shape from one station or the visibility of seismic phases across several stations, could easily be classified as earthquakes, some events were difficult to definitively classify as earthquakes.

Because the number of SLRNN detections not included in the catalog was not recorded, SLRNN detections from March 2022 were specifically analyzed to assess SLRNN precision in this study. The detection was tested for over one month with a network of 4 stations. With a detection threshold of at least two stations, 49 out of 462 detections were classified as earthquakes. Most earthquakes were identified based on clear signals with visible P and S phases at one station. Since the SLRNN does not pick seismic phases, many detections (initially reviewed manually) were microseismic events, which were later confirmed as real when reviewing EQTransformer detections. Applying EQTransformer detection to the one-month test period (March 2022) detects less than half of the events detected by SLRNN and verified manually.

## 4. Discussion

The problem of automatically detecting RTS events is complex and closely linked to the solutions provided by seismic networks. To study areas of low seismic activity with small-magnitude events, it is essential to detect weak tremors. However, the seismic network is often inconsistent and sparse, with too few stations, limiting the number of phase associations. The reduction in required picks increases the number of false detections (Table 4). The examined RTS cases yielded comparable detection results, regardless of the seismicity conditions or the automatic detection methods used, including EQTransformer, PhaseNet, PhaseNet with transfer learning, and the simpler SLRNN. A comparison of these algorithms reveals that no single method is sufficient for automating the detection of RTS, as indicated by the low F1 scores. At its maximum F1 score, the recall coefficient is approximately 80%. The number of extra detections then exceeds the number of positive detections several times (Figure 6 and Figure 7). While the maximum recall values, which approach one on the recall-precision diagrams, are unreliable, low precision increases the likelihood of coincidental time matches between automatically detected signals and cataloged earthquakes. Limiting the association to 4–5 picks, while requiring at least two S picks and peak values above 0.4–0.5, seems to produce reasonable results for further analysis. Nevertheless, automatic detections identify many events that are missed during manual processing. The study of additional detections revealed four similar groups of events across all RTS cases:

1. Missed Seismic Events: These events were obviously overlooked in the reference catalog. The manual catalog often reflects human error, such as missing specific periods or conducting superficial analyses, which is evident in the higher number of extra shocks recorded during the initial operation of the STr2 network. Omissions may be related to both periods of high seismic activity and periods of lower seismic activity.
2. Insufficiently Recorded Phenomena: Some phenomena resemble seismic events but are recorded at too few stations for reliable processing. In these cases, automatic detection often produces false picks at other stations. While it is possible to eliminate such detections during phase association, this requires an accurate velocity model.
3. Distant earthquakes: These events are not classified as RTS but were detected in all RTS cases.
4. False Detections: The occurrence of false detections, which resemble seismic events but are not reliably located, particularly regarding erroneous picks of S waves, hampers the automation of event detection. Consequently, these signals must be manually verified and corrected, as demonstrated using SLRNN in Lai Chau.

A comparison of EQTransformer detections with the catalog in Lai Chau, which includes phenomena detected by SLRNN, reveals that simpler detection methods can identify events overlooked by DNNs. SLRNN was developed for detection at a limited number of stations. The example from March 2022 in Lai Chau demonstrates the insufficient effectiveness of DNN compared to SLRNN. The available training data poses a challenge for training detection models due to the small number of phenomena from specific RTS regions. This limitation supports the use of simpler detection methods, such as SLRNN, which can function effectively with much smaller datasets than those required for DNNs. However, this is a very weak phenomenon. In the remaining cases of networks with a small number of stations (as the cases of STr2 in 2018, and Lau Chau in 2014), DNNs demonstrate exemplary performance. The low precision value for STr2 in 2018 can be attributed to the decay of seismicity typical for RTS.

The examined case demonstrated that transfer learning could improve the detectable performance of a model trained on one database but not on another, indicating that some training data used in SeisBench models do not differ significantly from the RTS. Transfer learning demonstrated good generalization across varying seismicity, allowing for its application to RTS. However, its effectiveness was not proven to be significant. Therefore, the application of STEAD or SCEDC training data from SeisBench would be a suitable starting point for automatic detection in RTS cases, particularly when the acquired data from the investigated network is insufficient for neural network training.

We did not observe an evident influence of non-stationarity on detection performance. In particular, the limited impact of transfer learning in some cases suggests that moderate changes in the training of seismic data are insufficient to produce significantly different detection results. Additionally, although we observe distinct time changes in seismicity at Song Tranh 2, the training dataset derived from periods of intense seismic activity does not degrade detection performance during periods of low seismicity.

Nevertheless, DNNs trained on natural shocks and SLRNNs trained on RTS phenomena can complement each other. Analyzing the detected events not included in the relevant catalog significantly increases the number of RTS research events. This expansion may facilitate retraining the detector to identify RTS events, potentially using a multi-stage training approach similar to that employed in the case of SLRNN.

**Figure 8:**
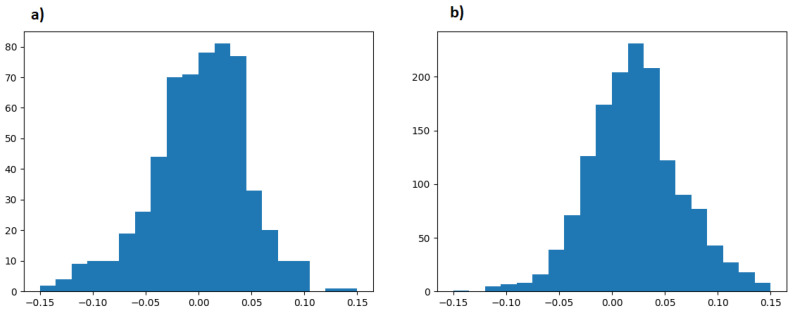
Histogram of differences between manual picking and automatic picking by EQTransformer pretrained on SCEDC dataset: (**a**) P waves and (**b**) S waves.

In this study, deep learning phase pickers were treated as seismic wave detectors. However, the phase association requires minimal phase time accuracy. The differences between automatic and manual picking times (Figure 8) are sufficient for phase association, especially since the alternative SLRNN phase detector assumes a delay of more than 0.1 s.

There are two key applications where automatic detection has proven useful during RTS analysis:

- Supporting Manual Analysis: Automatic detection reduces the need for extensive manual review of continuous recordings, acknowledging that many more detections than useful shocks require manual verification, which can lead to missing some events.
- Reanalysis of Undetected Phenomena: Automatic detection enables the reanalysis and identification of previously undetected phenomena. It can supplement previous analyses to enhance the capabilities of the RTS study.

The choice of the model must be tailored to the individual network and RTS case, as shown above.

## 5. Conclusions

The seismic signal detection models and training databases currently in use miss a noticeable percentage of events and should not be relied upon as the sole detection method. While retraining the network on local events could improve performance, this is limited by the availability of RTS events. The small and fluctuating number of continuously operational stations further complicates the situation. However, analyzing the additional detections of events not included in the reference catalog has shown that deep learning automatic detection can significantly increase the number of RTS events available for research. No degradation in detection performance was observed when training and detecting for non-stationary seismicity.

SLRNN performs better than the tested DNN detection at a smaller number of stations because it generates fewer false detections. It always detects S waves. Therefore, it is useful even for 2–3 stations, which is crucial for the studied RTS cases. However, it has much worse picking accuracy, which compromises the quality of phase association. It also misses some phenomena, and these detections complement each other.

ANNs enable the extraction of information from low-quality recordings under limited network geometry, reflecting the realities of seismological monitoring systems. The effectiveness of ANN-based algorithms does not come from their methodological superiority, but from existing network limitations. These, in turn, come from economic and logistical factors, over which we have limited influence. Network densification, whether achieved through surface or borehole stations, would be the best solution. However, it is always a trade-off between the optimal network configuration and what is feasible in remote areas with complex topography and limited access to power lines and other required media. Both automatic and manual detection methods struggle to identify seismic events recorded poorly and at insufficient stations, which limits further analysis and interpretation. An acceptable recall is associated with significantly inferior precision. A well-chosen dense seismic network, rather than just a detection method, is essential for automatically detecting and processing weak seismic events.

## Figures and Tables

**Table 1:** The current datasets of the RTS areas.

Dataset	Events	P Picks	S Picks	Magnitude Ranges
Song Tranh 2	7654	31,713	34,148	−0.6 to 3.6
Lau Chau	2048	8572	8046	0.1 to 5.1
Czorsztyn	298	1884	1930	−0.7 to 3.2

**Table 2:** Overview of the datasets applied to STr2 [39].

Dataset	Number of Records	Number of Events	Number of P_Picks	Number of S_Picks	Number of Noise	Region
LenDB	1,244,942	303,902	629,095	0	615,847	various
SCEDC	8,111,060	378,528	7,571,970	4,364,155	0	Southern California
STEAD	1,265,657	441,705	1,030,231	1,030,231	235,426	various

**Table 3:** Event detection performance metrics for the PhaseNet network trained with global database models and transfer learning on the STr2 database. The phase threshold was 0.4, and 4 picks were required for association by PyOcto.

Model	Years								
	2013			2014			2018		
	Recall	Precision	F1	Recall	Precision	F1	Recall	Precision	F1
SCEDC	0.96	0.36	0.53	0.96	0.31	0.46	0.91	0.01	0.02
SCEDC transferred to Str2	0.94	0.31	0.47	0.95	0.30	0.45	0.89	0.01	0.01
STEAD	0.73	0.57	0.64	0.82	0.53	0.64	0.94	0.01	0.03
STEAD transferred to Str2	0.98	0.33	0.50	0.97	0.31	0.47	0.94	0.01	0.01

**Table 4:** Event detection results in Lai Chau.

Year	Required Phases	Min. Peak Value	Extra Detections	Local Events in Extra Detections	Local Events Visible at min. 4 Stations	False Detections	Other Extra Detections
2014	4	0.8	4	4	1	0	0
		0.7	6	6	2	0	0
		0.6	16	16	4	0	0
		0.5	38	34	5	2	2
		0.4	81	64	7	5	16
	3	0.9	1	1	1	0	0
		0.8	18	17	7	1	0
		0.7	42	39	12	2	1
		0.6	82	71	16	5	6
2018	5	0.8	3	1	1	0	2
		0.7	15	11	6	1	3
		0.6	75	65	30	3	9
	4	0.9	1	1	0	0	0
		0.8	15	13	9	0	2
		0.7	73	68	28	0	7

## Data Availability

The SWIP5 program is available at https://github.com/JanWiszniowski/SWIP5 (accessed on 14 December 2025). The SLRNN plugin for the SWIP5 program is available at https://github.com/JanWiszniowski/SLRNN (accessed on 14 December 2025). SeisBench is available at https://github.com/seisbench/seisbench (accessed on 14 December 2025), PhaseNet is available at https://github.com/AI4EPS/PhaseNet (accessed on 14 December 2025), and PyOcto is available at https://github.com/yetinam/pyocto (accessed on 14 December 2025). Catalogs of seismic events, information about stations, and waveforms from Czorsztyn, Song Tranh2, and Lai Chau are available in the EPISODES platform under the following links: https://episodesplatform.eu/?lang=en#episode:CZORSZTYN (accessed on 10 December 2025), https://episodesplatform.eu/?lang=en#episode:SONG_TRANH (accessed on 10 December 2025), https://episodesplatform.eu/?lang=en#episode:LAI_CHAU (accessed on 10 December 2025). The above datasets are open under a Creative Commons license.

## Abbreviations

The following abbreviations are used in this manuscript:

RTS	Reservoir-triggered seismicity
DNN	Deep neural networks
SLRNN	Single-layer recurrent neural network
STr2	Song Tranh 2
IG PAS	Institute of Geophysics, Polish Academy of Sciences
IGP VAST	Institute of Geophysics, Vietnam Academy of Science and Technology
CNN	Convolutional neural network
GPD	Generalized Seismic Phase Detector
SCEDC	The Southern Californian Earthquake Data Centre dataset
STEAD	Stanford earthquake dataset
TP	True positive
FP	False positive
FN	False negative
TN	True negative
R	Recall
P	Precision

## References

[1] Gupta H.K. (2022). Artificial Water Reservoir-Triggered Seismicity (RTS): Most Prominent Anthropogenic Seismicity. Surv. Geophys..

[2] Carder D.S. (1945). Seismic investigations in the Boulder Dam area, 1940–1944, and the influence of reservoir loading on earthquake activity. Bull. Seismol. Soc. Am..

[3] Gupta H.K. (2002). A review of recent studies of triggered earthquakes by artificial water reservoirs with special emphasis on earthquakes in Koyna, India. Earth Sci. Rev..

[4] Fan Z., Eichhubl P., Newell P. (2019). Basement fault reactivation by fluid injection into sedimentary reservoirs: Poroelastic effects. J. Geophys. Res. Solid Earth.

[5] Büyükakpınar P., Cesca S., Hainzl S., Jamalreyhani M., Heimann S., Dahm T. (2021). Reservoir-Triggered Earthquakes Around the Atatürk Dam (Southeastern Turkey). Front. Earth Sci..

[6] Orlecka-Sikora B., Rudziński Ł., Staszek M., Lizurek G., Mizerski K. (2023). Seismic swarms as intermittent quasi-static ruptures driven by pore pressure variations due to the water reservoir impoundment. Tectonophysics.

[7] Yang Z., Yehya A., Iwalewa T.M., Rice J.R. (2021). Effect of permeability evolution in fault damage zones on earthquake recurrence. J. Geophys. Res. Solid Earth.

[8] Białoń W., Zarzycka E., Lasocki S. (2015). Seismicity of Czorsztyn Lake Region: A Case of Reservoir Triggered Seismic Process?. Acta Geophys..

[9] Białoń W., Lizurek G., Dec J., Cichostępski K., Pietsch K. (2019). Relocation of Seismic Events and Validation of Moment Tensor Inversion for SENTINELS Local Seismic Network. Pure Appl. Geophys..

[10] Wiszniowski J., Giang N.V., Plesiewicz B., Lizurek G., Van D.Q., Khoi L.Q., Lasocki S. (2015). Preliminary results of anthropogenic seismicity monitoring in the region of Song Tranh 2 Reservoir, Central Vietnam. Acta Geophys..

[11] Lizurek G., Wiszniowski J., Van Giang N., Plesiewicz B., Van D.Q. (2017). Clustering and Stress Inversion in the Song Tranh 2 Reservoir, Vietnam. Bull. Seismol. Soc. Am..

[12] Lizurek G., Wiszniowski J., Giang N.V., Van D.Q., Dung L.V., Tung V.D., Plesiewicz B. (2019). Background seismicity and seismic monitoring in the Lai Chau reservoir area. J. Seismol..

[13] Tung N.T. (1996). The induced seismicity at Hoa Binh Reservoir region. Abstract Vol. IASPEI Reg. Assembly in Asia.

[14] Withers M., Aster R., Young C., Beriger J., Harris M., Moore S., Trujillo J. (1998). A Comparison of Select Trigger Algorithms for Automated Global Seismic Phase and Event Detection. Bull. Seismol. Soc. Am..

[15] Allen R. (1978). Automatic earthquake recognition and timing from single traces. Bull. Seismol. Soc. Am..

[16] Allen R. (1982). Automatic phase pickers: Their present use and future prospects. Bull. Seismol. Soc. Am..

[17] Li J., Stankovic L., Stankovic V., Pytharouli S., Yang C., Shi Q. (2023). Graph-Based Feature Weight Optimisation and Classification of Continuous Seismic Sensor Array Recordings. Sensors.

[18] Romeo G. (1994). Seismic signals detection and classification using artiricial neural networks. Ann. Geophys..

[19] Wang J., Teng T.-L. (1995). Artificial neural network-based seismic detector. Bull. Seismol. Soc. Am..

[20] Tiira T. (1999). Detecting teleseismic events using artificial neural networks. Comput. Geosci..

[21] Fradkov A.L. (2020). Early History of Machine Learning. IFAC-Pap..

[22] Yu S., Ma J. (2021). Deep learning for geophysics: Current and future trends. Rev. Geophys..

[23] Mousavi S.M., Beroza G.C. (2023). Machine Learning in Earthquake Seismology. Annu. Rev. Earth Planet. Sci..

[24] Kubo H., Naoi M., Kano M. (2024). Recent advances in earthquake seismology using machine learning. Earth Planets Space.

[25] Aguilar Suarez A.L., Beroza G. (2025). Picking Regional Seismic Phase Arrival Times with Deep Learning. Seismica.

[26] Jiang C., Fang L.H., Fan L.P., Li B.R. (2021). Comparison of the earthquake detection abilities of PhaseNet and EQTransformer with the Yangbi and Maduo earthquakes. Earthq. Sci..

[27] Park Y., Beroza G.C., Ellsworth W.L. (2023). A Mitigation Strategy for the Prediction Inconsistency of Neural Phase Pickers. Seismol. Res. Lett..

[28] Niksejel A., Zhang M. (2024). OBSTransformer: A Deep-Learning Seismic Phase Picker for OBS Data Using Automated Labelling and Transfer Learning. Geophys. J. Int..

[29] Chai C., Maceira M., Santos-Villalobos H.J., Venkatakrishnan S.V., Schoenball M., Zhu W., Beroza G.C., Thurber C., EGS Collab Team (2020). Using a deep neural network and transfer learning to bridge scales for seismic phase picking. Geophys. Res. Lett..

[30] Thuy N.N., Phach P.V., Chinh V.V., Minh L.H., Nguyen P.D., Hung P.Q., Duong N.A. (2003). Report on estimation of the seismic design for the Song Tranh 2 hydropower construction. Archives of the Institute of Geophysics.

[31] Plesiewicz B., Wiszniowski J., Karkowska K., Kijko A. (2024). Seismic hazard assessment in the Podhale region, Poland—Zone and smoothed seismicity approach. Acta Geophys..

[32] Staszek M., Plesiewicz B., Rudziński Ł., Lizurek G., Czerwiński T., Musiatewicz M., Karkowska K., Michałowski K. (2024). Natural seismicity of Poland complemented by the results of Geodynamic Monitoring of Poland project (2013–2023). Prz. Geol..

[33] Trojanowski J., Plesiewicz B., Wiszniowski J. (2015). Seismic monitoring of Poland—Temporary Seismic Project with mobile Seismic Network. Acta Geophys..

[34] Wiszniowski J., Plesiewicz B.M., Trojanowski J. (2014). Application of real time recurrent neural network for detection of small natural earthquakes in Poland. Acta Geophys..

[35] Doubravová J., Wiszniowski J., Horálek J. (2016). Single Layer Recurrent Neural Network for detection of swarm-like earthquakes in W-Bohemia/Vogtland—The method. Comput. Geosci..

[36] Wiszniowski J., Plesiewicz B., Lizurek G. (2021). Machine learning applied to anthropogenic seismic events detection in Lai Chau reservoir area, Vietnam. Comput. Geosci..

[37] Wiszniowski J. (2000). Application of real time recurrent neural network for seismic event detection. Acta Geophys. Pol..

[38] Narendra K., Parthasarathy K. (1991). Gradient methods for the optimization of dynamical systems containing neural networks. IEEE Trans. Neural Netw..

[39] Woollam J., Münchmeyer J., Tilmann F., Rietbrock A., Lange D., Bornstein T., Diehl T., Giuchi C., Haslinger F., Jozinović D. (2022). SeisBench—A Toolbox for Machine Learning in Seismology. Seismol. Res. Lett..

[40] Woollam J., Rietbrock A., Bueno A., Angelis S. (2019). Convolutional neural network for seismic phase classification, performance demonstration over a local seismic network. Seismol. Res. Lett..

[41] Mousavi S.M., Ellsworth W.L., Zhu W., Chuang L.Y., Beroza G.C. (2020). Earthquake transformer—An attentive deep-learning model for simultaneous earthquake detection and phase picking. Nat. Commun..

[42] Ross Z.E., Meier M.-A., Hauksson E., Heaton T.H. (2018). Generalized seismic phase detection with deep learning. Bull. Seismol. Soc. Am..

[43] Zhu W., Beroza G.C. (2019). Phasenet: A deep-neural-network-based seismic arrival-time picking method. Geophys. J. Int..

[44] Magrini F., Jozinović D., Cammarano F., Michelini A., Boschi L. (2020). Local earthquakes detection: A benchmark dataset of 3-component seismograms built on a global scale. Artif. Intell. Geosci..

[45] SCEDC (2013). Southern California Earthquake Center. Caltech. Dataset. https://scedc.caltech.edu/.

[46] Mousavi S.M., Sheng Y., Zhu W., Beroza G.C. (2019). Stanford earthquake dataset (stead): A global data set of seismic signals for AI. IEEE Access.

[47] Ross Z.E., Yue Y., Meier M.-A., Hauksson E., Heaton T.H. (2019). PhaseLink: A deep learning approach to seismic phase association. J. Geophys. Res. Solid Earth.

[48] Münchmeyer J. (2024). PyOcto: A high-throughput seismic phase associator. Seismica.

[49] Rudziński Ł., Lasocki S., Orlecka-Sikora B., Wiszniowski J., Olszewska D., Kokowski J., Mirek J. (2021). Integrating Data under the European Plate Observing System from the regional and selected local seismic network in Poland. Seism. Res. Lett..

